# Supplementary material for: PTEN self-regulates through USP11 via the PI3K-FOXO pathway to stabilize tumor suppression
Source: Nat Commun. 2019 Feb 7;10:636. doi: 10.1038/s41467-019-08481-x (PMC6367354; doi:10.1038/s41467-019-08481-x)
Supplement: Supplementary file 1 — Supplementary Information [file 41467_2019_8481_MOESM1_ESM.pdf]

Supplementary Information

PTEN self-regulates through USP11 via the PI3K-FOXO pathway to stabilize tumor suppression

Park et al.

Supplementary Figures

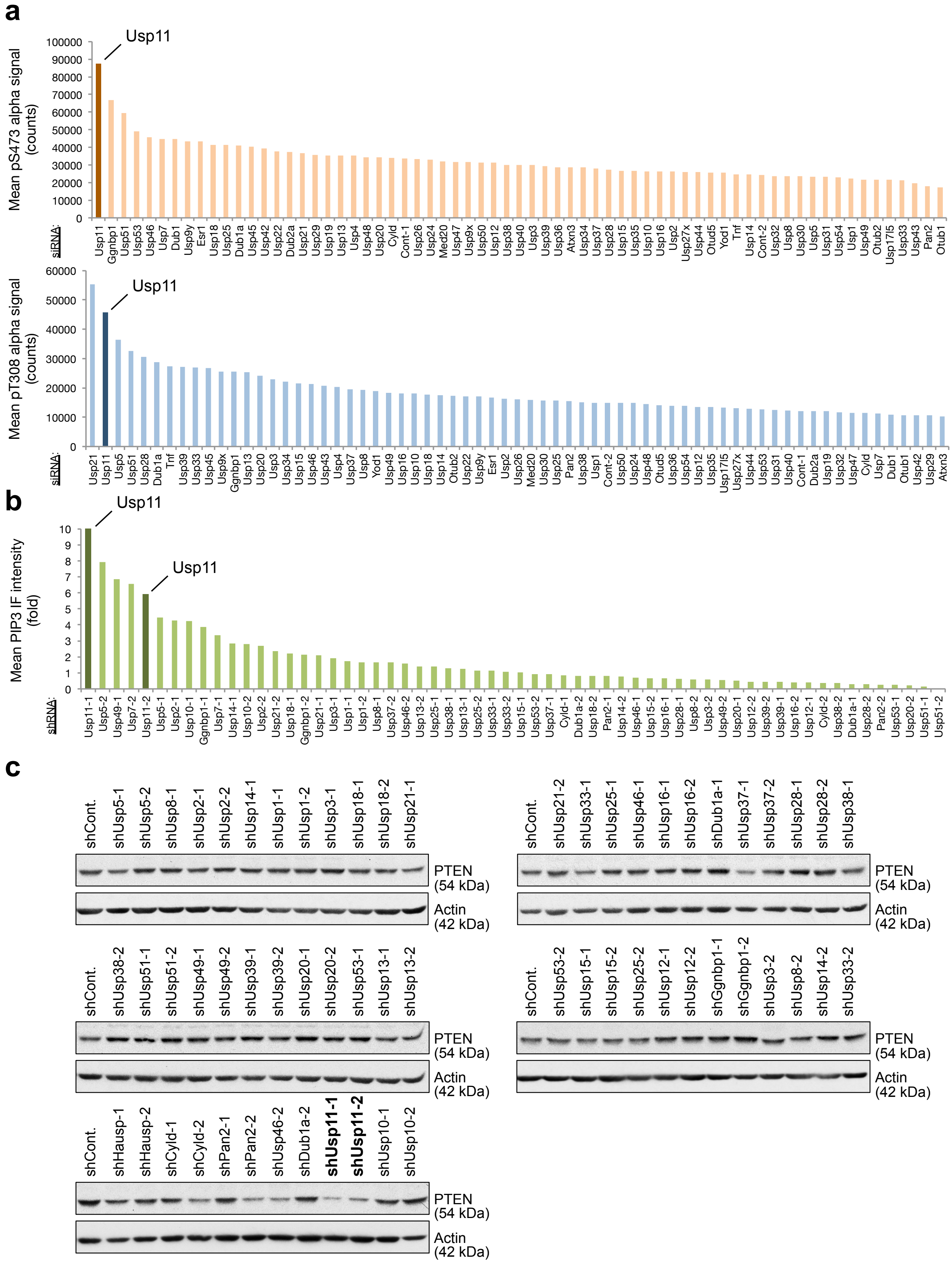

Supplementary Figure 1. An RNAi screen identifies USP11 as a deubiquitinase that regulates PTEN protein levels and activity

(**a**) Mouse embryonic fibroblasts (MEFs) transfected with a synthetic siRNA library targeting 67 mouse DUBs for 72 h were subjected to the screen for phosphorylation of AKT using P-AKT (pS473 (top) and pT308 (bottom)) AlphaScreen assays.

(**b**) Immunofluorescence (IF) analysis of PIP3 in MEFs expressing two individual clones of 30 DUB shRNAs chosen from (**a**), starved for 8 h and stimulated with 100 nM insulin for 5 min. The PIP3 IF intensity was determined by the ImageJ 1.46r software and normalized to that in MEFs expressing control shRNA.

(**c**) Lysates from MEFs expressing two individual clones of 30 DUB shRNAs chosen from (**a**) were subjected to immunoblotting.

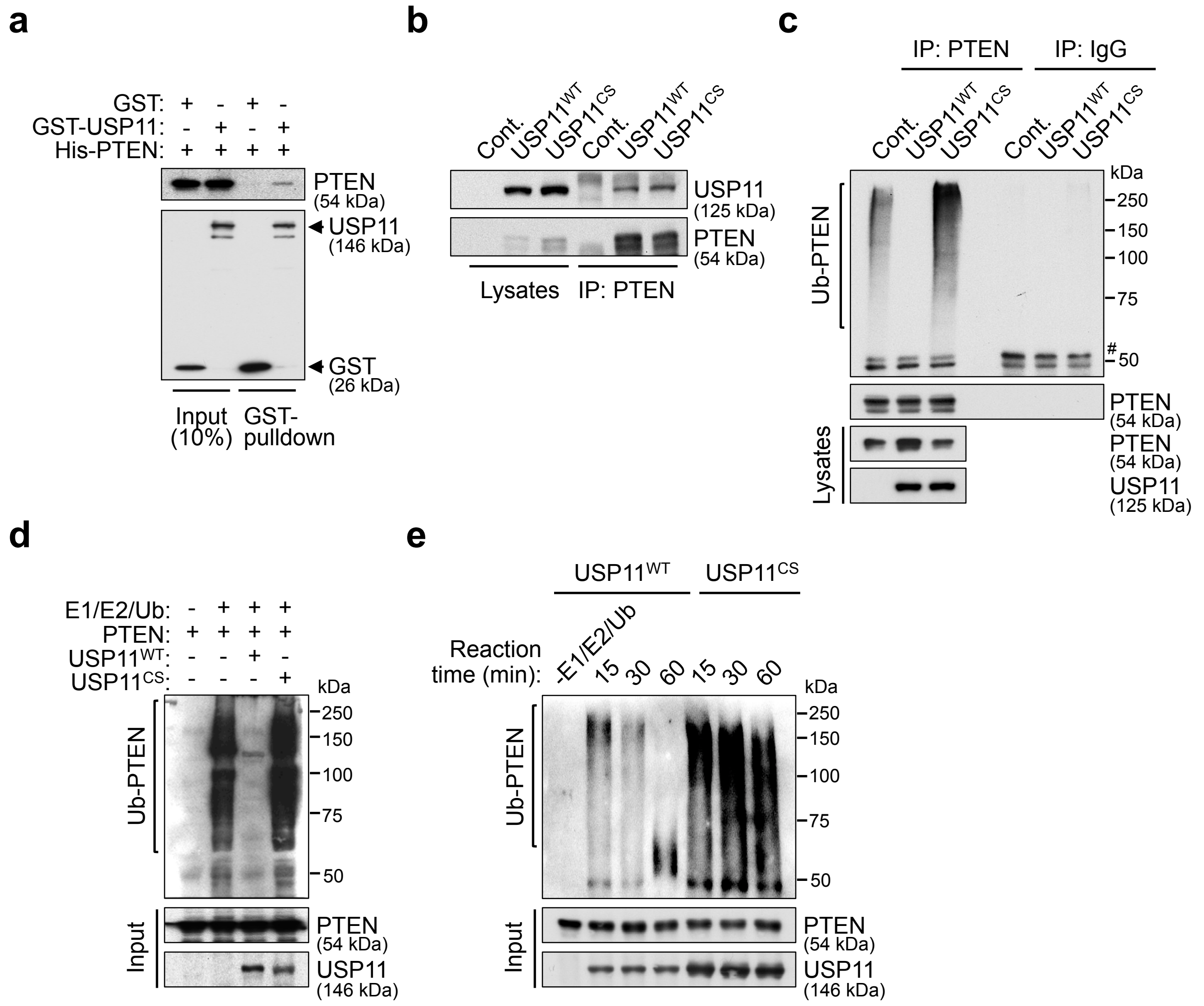

Supplementary Figure 2. USP11 directly interacts with and deubiquitinates PTEN

(**a**) The recombinant glutathione S-transferase (GST)–USP11 proteins were pull-downed with His-PTEN proteins, and the beads were then washed, eluted and subjected to immunoblotting (IB).

(**b**) Lysates from 293T cells expressing Flag-tagged wild-type (WT) or C318S (CS) mutant USP11 were immunoprecipitated with anti-Myc-PTEN then immunoblotted.

(**c**) Lysates from DU145 cells overexpressing WT or CS mutant USP11 and treated with 10 µM MG132 for 4 h were IP with anti-PTEN, and the resulting immunoprecipitates were subjected to IB. # indicates heavy chain of IgG.

(**d**) His-PTEN proteins were incubated with recombinant WT or CS mutant USP11 in the

presence of ubiquitin, E1, E2 (UbcH5) and 293T cell extracts (1 μl), and the resulting ubiquitin-conjugated PTEN proteins were subjected to metal-affinity purification then IB. (**e**) Time course of deubiquitination of ubiquitin-conjugated PTEN proteins by WT or CS mutant USP11. His-PTEN proteins were incubated with recombinant WT or CS mutant USP11 in the presence of ubiquitin, E1, E2 (UbcH5) and 293T cell extracts (1 μl) for the indicated times, and the resulting ubiquitin-conjugated PTEN proteins were subjected to metal-affinity purification then IB.

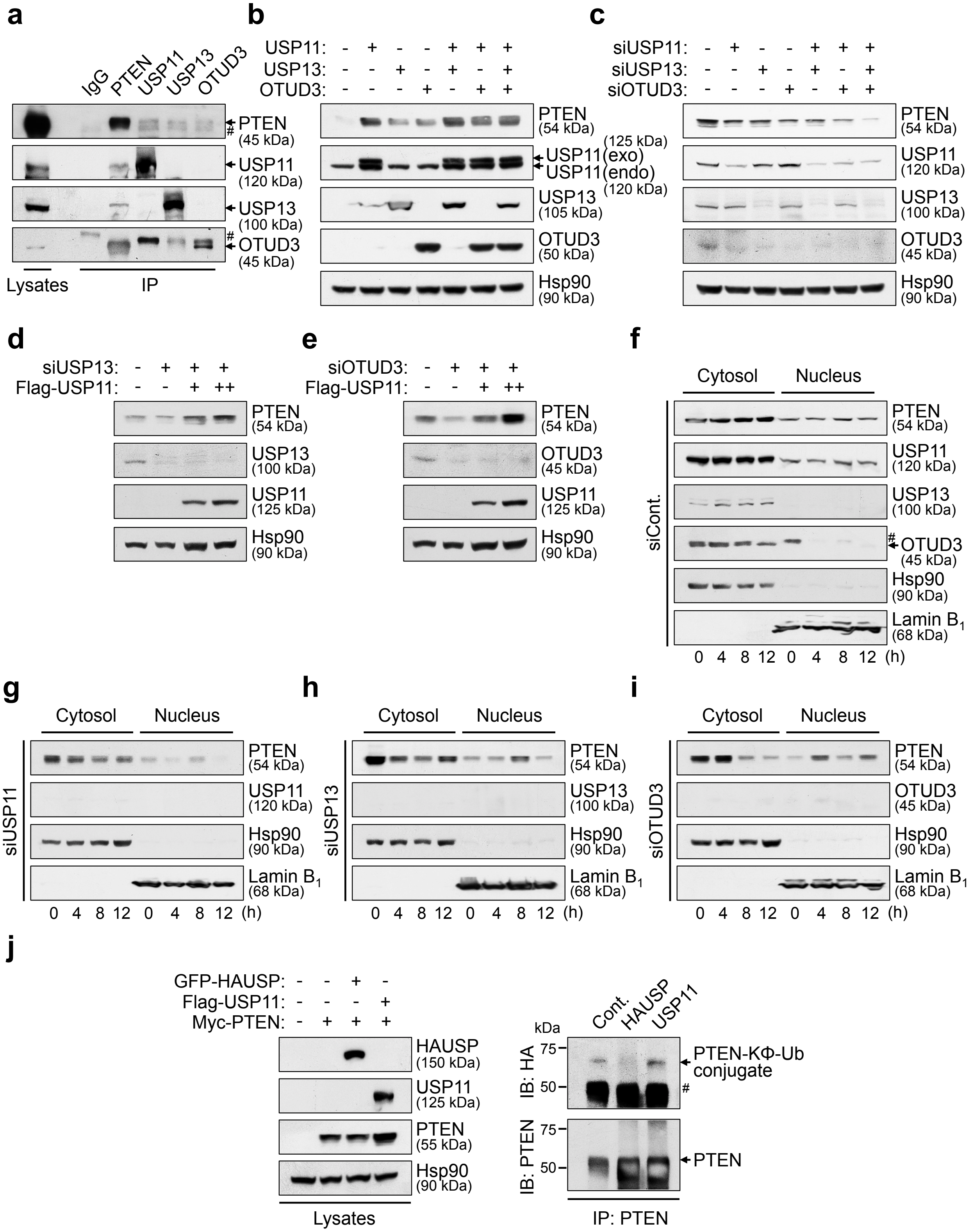
a
IgG
PTEN
USP11
USP13
OTUD3
PTEN (45 kDa)
USP11 (120 kDa)
USP13 (100 kDa)
OTUD3 (45 kDa)
Lysates
IP
b
USP11:
USP13:
OTUD3:
PTEN (54 kDa)
USP11(exo) (125 kDa)
USP11(endo) (120 kDa)
USP13 (105 kDa)
OTUD3 (50 kDa)
Hsp90 (90 kDa)
c
siUSP11:
siUSP13:
siOTUD3:
PTEN (54 kDa)
USP11 (120 kDa)
USP13 (100 kDa)
OTUD3 (45 kDa)
Hsp90 (90 kDa)
d
siUSP13:
Flag-USP11:
PTEN (54 kDa)
USP13 (100 kDa)
USP11 (125 kDa)
Hsp90 (90 kDa)
e
siOTUD3:
Flag-USP11:
PTEN (54 kDa)
OTUD3 (45 kDa)
USP11 (125 kDa)
Hsp90 (90 kDa)
f
Cytosol
Nucleus
siCont.
PTEN (54 kDa)
USP11 (120 kDa)
USP13 (100 kDa)
OTUD3 (45 kDa)
Hsp90 (90 kDa)
Lamin B1 (68 kDa)
0 4 8 12 0 4 8 12 (h)
g
Cytosol
Nucleus
siUSP11
PTEN (54 kDa)
USP11 (120 kDa)
Hsp90 (90 kDa)
Lamin B1 (68 kDa)
0 4 8 12 0 4 8 12 (h)
h
Cytosol
Nucleus
siUSP13
PTEN (54 kDa)
USP13 (100 kDa)
Hsp90 (90 kDa)
Lamin B1 (68 kDa)
0 4 8 12 0 4 8 12 (h)
i
Cytosol
Nucleus
siOTUD3
PTEN (54 kDa)
OTUD3 (45 kDa)
Hsp90 (90 kDa)
Lamin B1 (68 kDa)
0 4 8 12 0 4 8 12 (h)
j
GFP-HAUSP:
Flag-USP11:
Myc-PTEN:
HAUSP (150 kDa)
USP11 (125 kDa)
PTEN (55 kDa)
Hsp90 (90 kDa)
Lysates
kDa
Cont.
HAUSP
USP11
IB: HA
75
50
PTEN-KΦ-Ub conjugate
IB: PTEN
75
50
PTEN
IP: PTEN

Supplementary Figure 3. USP11 is a unique DUB that de-polyubiquitinates and stabilizes PTEN protein in the nucleus

(**a**) Lysates from DU145 cells were immunoprecipitated (IP) with IgG control, PTEN, USP11, USP13 and OTUD3, as indicated, and subjected to immunoblotting (IB). # indicate heavy chain of IgG.

(**b**) Lysates from DU145 cells expressing control vector, Flag-USP11, Myc-USP13 and Myc-OTUD3, as indicated, were subjected to IB.

(**c**) Lysates from DU145 cells expressing non-targeting control, USP11, USP13 and OTUD3 siRNAs, as indicated, were subjected to IB.

(**d, e**) Lysates from DU145 cells expressing control vector or Flag-USP11 and USP13 (**d**) or OTUD3 (**e**) siRNAs, as indicated, were subjected to IB.

(**f–i**) Lysates from cytosolic and nuclear fractionation in DU145 cells expressing non-targeting control, USP11, USP13 and OTUD3 siRNAs, as indicated, were subjected to IB. Hsp90 and Lamin B_1 serve as loading controls. * indicates non-specific band.

(**j**) Lysates from PC3 cells expressing control vector, GFP-HAUSP, Flag-USP11 and Myc-PTEN, as indicated, in the presence of HA-KΦ-Ub were IP with PTEN, and then analyzed for mono-ubiquitination by IB with anti-HA. # indicates heavy chain of IgG.

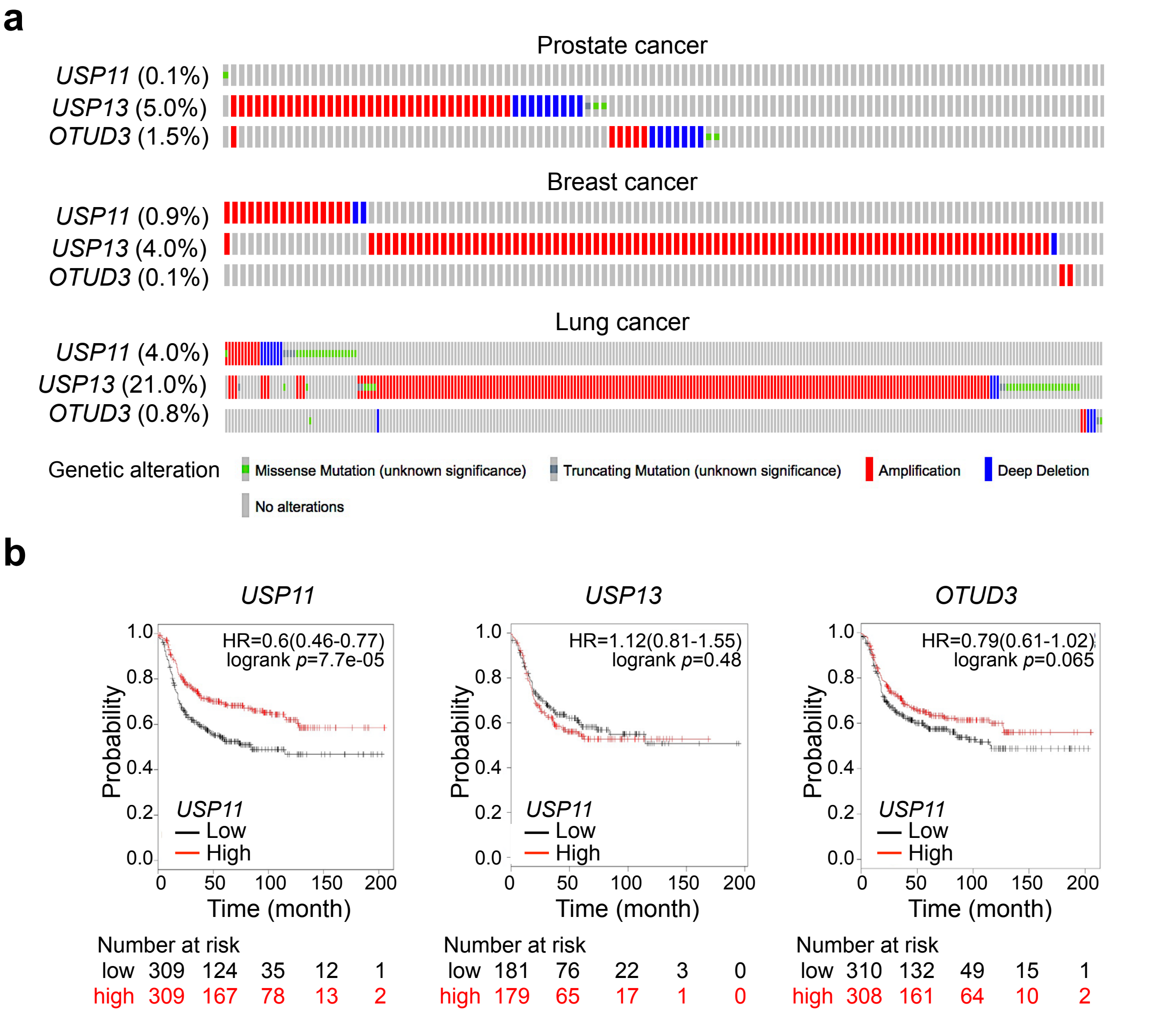

Supplementary Figure 4. Comparison of clinical relevance of *USP11*, *USP13* and *OTUD3* in human cancer patients.

(**a**) Genetic alterations of *USP11*, *USP13* and *OTUD3* in human prostate (n = 1013), breast (n = 2051) and lung (n = 1144) cancer patients.

(**b**) Online analysis of relapse-free survival (RFS) in human basal-type breast cancer patients with high or low *USP11* (n = 618), *USP13* (n = 360) or *OTUD3* (n = 618) expression. The number of surviving patients at different time points is indicated below the graphs. *p* value was determined by Log-rank (Mantel-Cox) test. HR, hazard ratio.

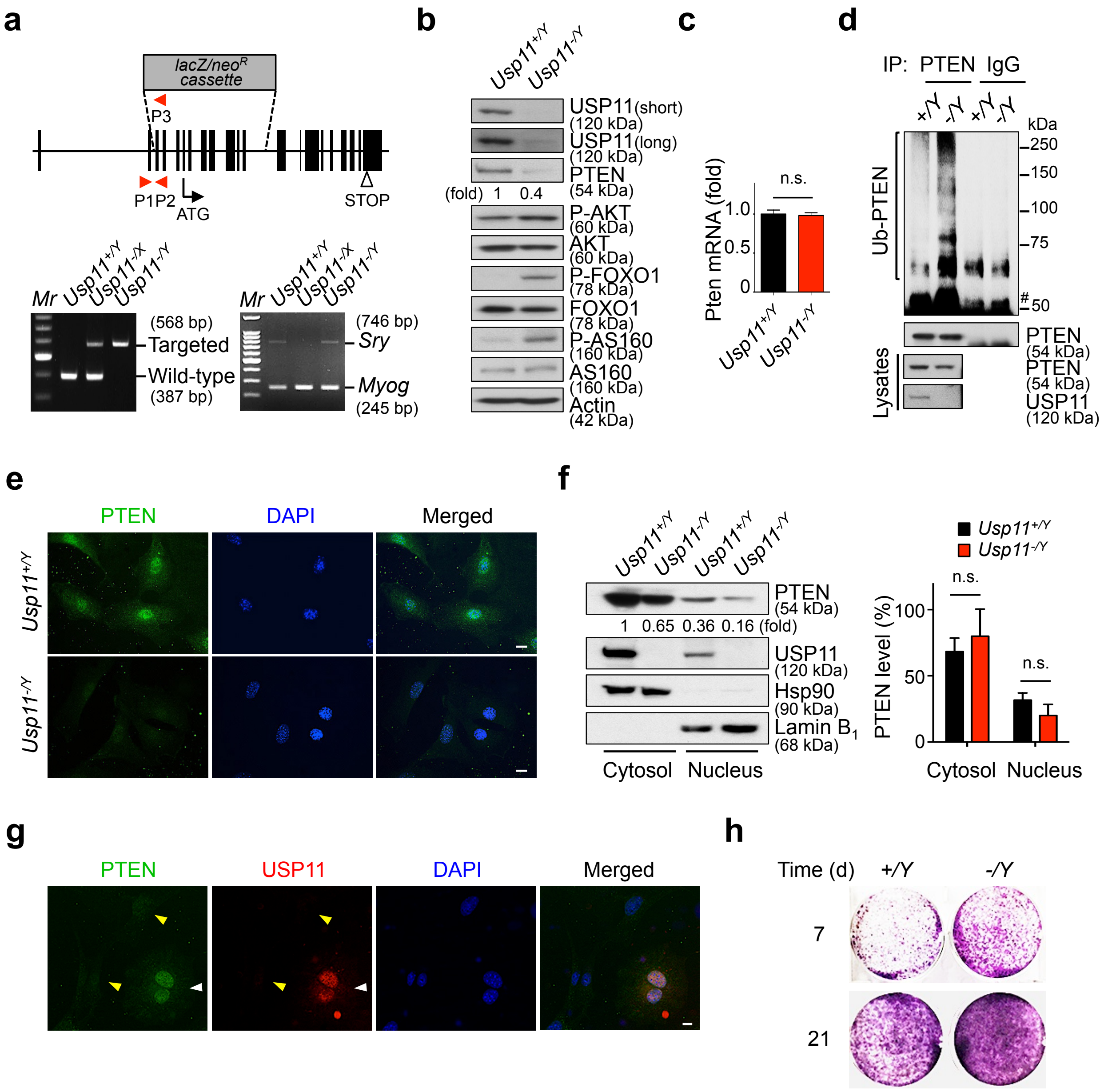

Supplementary Figure 5. USP11 regulates the PTEN protein level but not its subcellular localization

(**a**) Generation of *Usp11* knockout mice. A diagram depicting the gene-trapped allele of *Usp11* (top). Arrowheads indicate the position of genotyping primers (P1, P2 and P3). Genotype- (bottom, left) and sex– (bottom, right) specific PCR analysis of E13.5 embryos from an intercross of *Usp11*$^{-/Y}$ male and *Usp11*$^{+/-}$ female mice. The male-specific marker SRY (appears in the sex determination region on the Y chromosome)

and the universal marker myogenin (Myog) (expressed in both males and females) were used for sex determination.

(**b, c**) Lysates and total RNAs from primary wild-type (*Usp11*$^{+/Y}$) and *Usp11*$^{-/Y}$ MEFs were subjected to immunoblotting (IB) (**b**) and RT-qPCR (**c**). n = 3.

(**d**) Lysates from *Usp11*$^{+/Y}$ or *Usp11*$^{-/Y}$ MEFs treated with 10 μM MG132 for 4 h were immunoprecipitated (IP) with anti-PTEN, and the resulting IP were subjected to IB. # indicates heavy chain of IgG.

(**e, f**) Immunofluorescence analysis (**e**) and cytosolic and nuclear fractionation (**f**) of PTEN protein in primary *Usp11*$^{+/Y}$ and *Usp11*$^{-/Y}$ MEFs. The percentage of PTEN proteins detected in cytosolic and nuclear fractionation was also quantified. Hsp90 and Lamin B_1 serve as controls. Scale bars, 10 μm. n = 3.

(**g**) Immunofluorescence analysis of PTEN protein in primary *Usp11*$^{+/Y}$ and *Usp11*$^{-/Y}$ MEFs simultaneously seeded on the same well. White and yellow arrowheads indicate *Usp11*$^{+/Y}$ and *Usp11*$^{-/Y}$ MEFs, respectively. Scale bars, 10 μm.

(**h**) Time course of colony-formation efficiency of primary *Usp11*$^{+/Y}$ and *Usp11*$^{-/Y}$ MEFs. Error bars represent +/- SEM. *p* value was determined by Student's *t* test (n.s., non-significant).

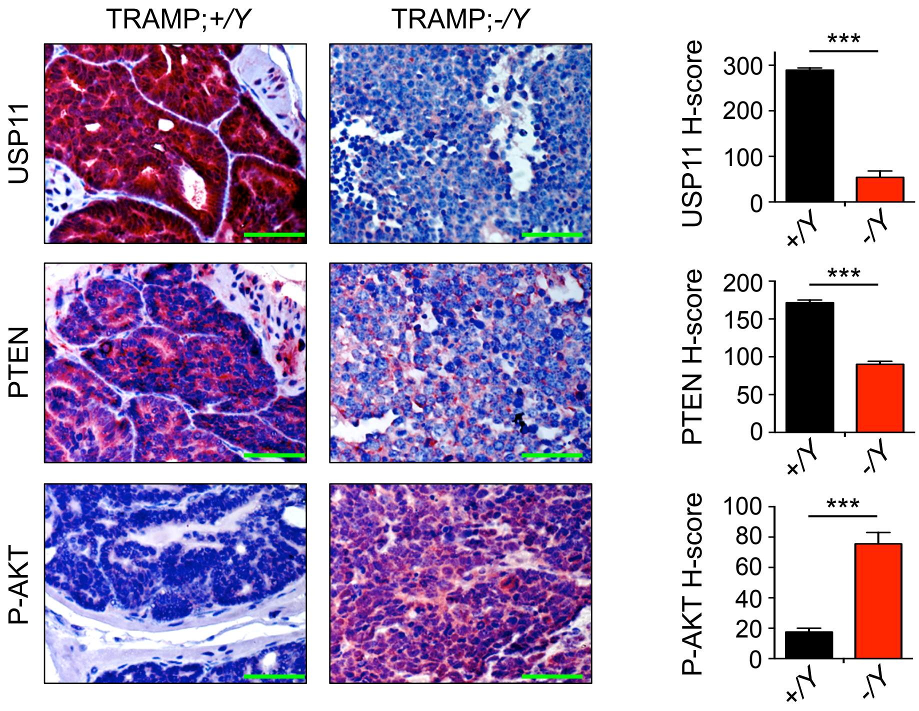

Supplementary Figure 6. *Usp11* ablation reduces PTEN expression in TRAMP mice

Sections of prostate isolated from 25-week-old TRAMP;*Usp11*$^{+/Y}$ and TRAMP;*Usp11*$^{-/Y}$ mice stained with anti-USP11, anti-PTEN and anti-P-AKT (left). Quantification of the number of immunohistochemical analysis is shown in right. Scale bars, 75 μm. n = 4. Error bars represent +/- SEM. *p* value was determined by Student's *t* test (**p*<0.05; ***p*<0.01; ****p*<0.001).

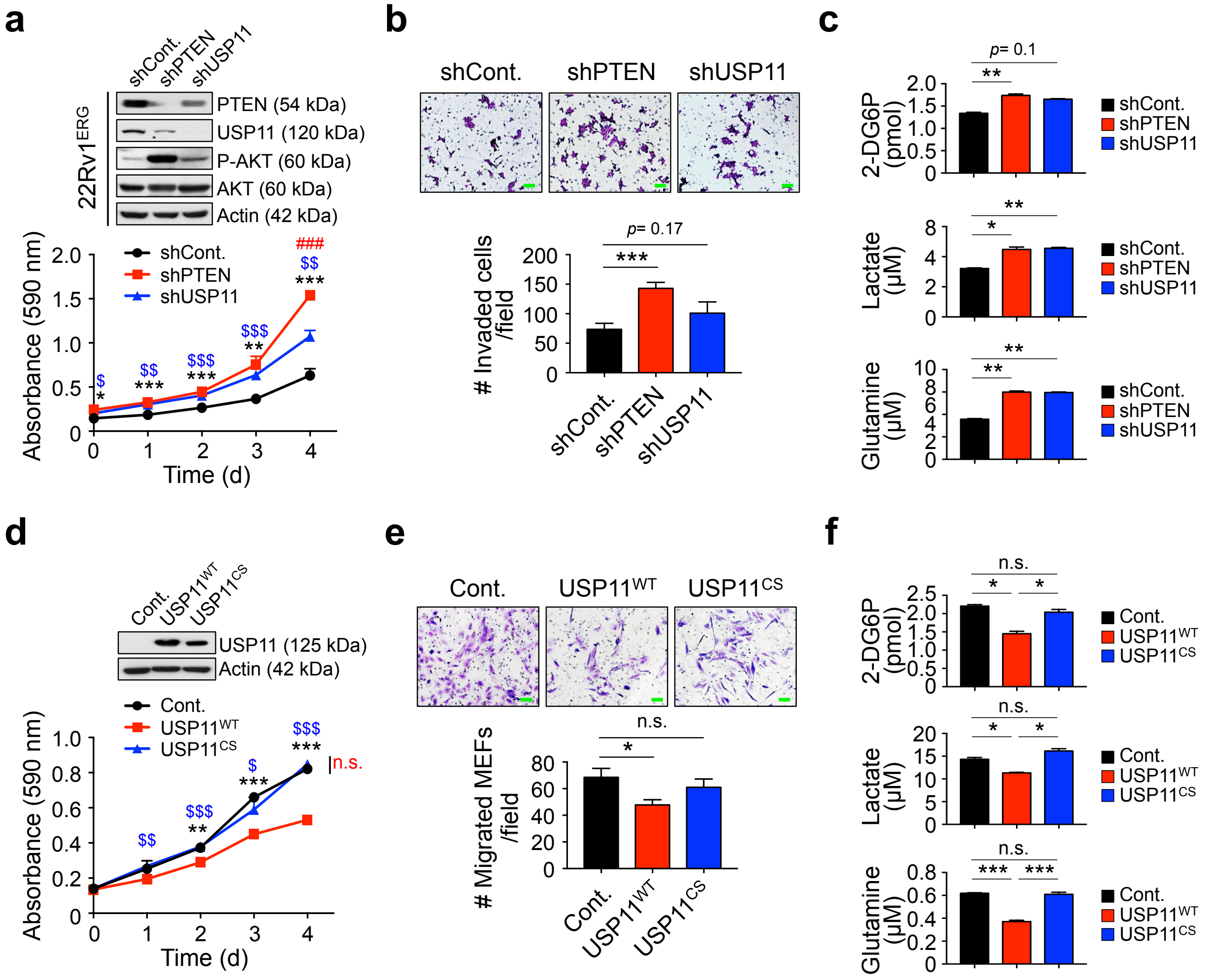

Supplementary Figure 7. DUB activity by USP11 is essential to USP11-mediated tumor suppression

(**a**) Growth curves of 22Rv1^{ERG} cells ectopically expressing PTEN or USP11 shRNA. n = 3. *p* value was determined by Student's *t* test ($^{*}p<0.05$, $^{**}p<0.01$, $^{***}p<0.001$, shCont. vs. shPTEN; $^{\$}p<0.05$, $^{\$\$}p<0.01$, $^{\$\$\$}p<0.001$, shCont. vs. shUSP11; $^{\#\#\#}p<0.01$, shPTEN vs. shUSP11).

(**b**) Cell invasion assays of 22Rv1^{ERG} cells ectopically expressing PTEN or USP11 shRNA in the presence of mitomycin C (5 μg ml^{-1}) (top). The number of invaded cells per field was quantified (bottom). n = 3.

(**c**) The rates of glucose uptake, lactate production and glutamine consumption of 22Rv1^{ERG} cells ectopically expressing PTEN or USP11 shRNA were measured and normalized to cell number. n = 3.

(**d**) Growth curves of *Usp11*$^{-/Y}$ MEFs expressing wild-type (WT) or catalytically inactive C318S (CS) mutant of USP11. n = 3. *p* value was determined by Student's *t* test (***p*<0.01, ****p*<0.001, Cont. vs. USP11^{WT}; $^{\$}p<0.05$, $^{\$\$}p<0.01$, $^{\$\$\$}p<0.001$, USP11^{WT} vs. USP11^{CS}; n.s., non-significant, Cont. vs. USP11^{CS}).

(**e**) Cell invasion assays of *Usp11*$^{-/Y}$ MEFs expressing WT or CS mutant of USP11 in the presence of mitomycin C (5 μg ml^{-1}) (top). The number of invaded cells per field was quantified (bottom). n = 3.

(**f**) The rates of glucose uptake, lactate production and glutamine consumption of *Usp11*$^{-/Y}$ MEFs expressing WT or CS mutant of USP11 were measured and normalized to cell number. n = 3.

Error bars represent +/- SEM. *p* value was determined by Student's *t* test (**p*<0.05; ***p*<0.01; ****p*<0.001).

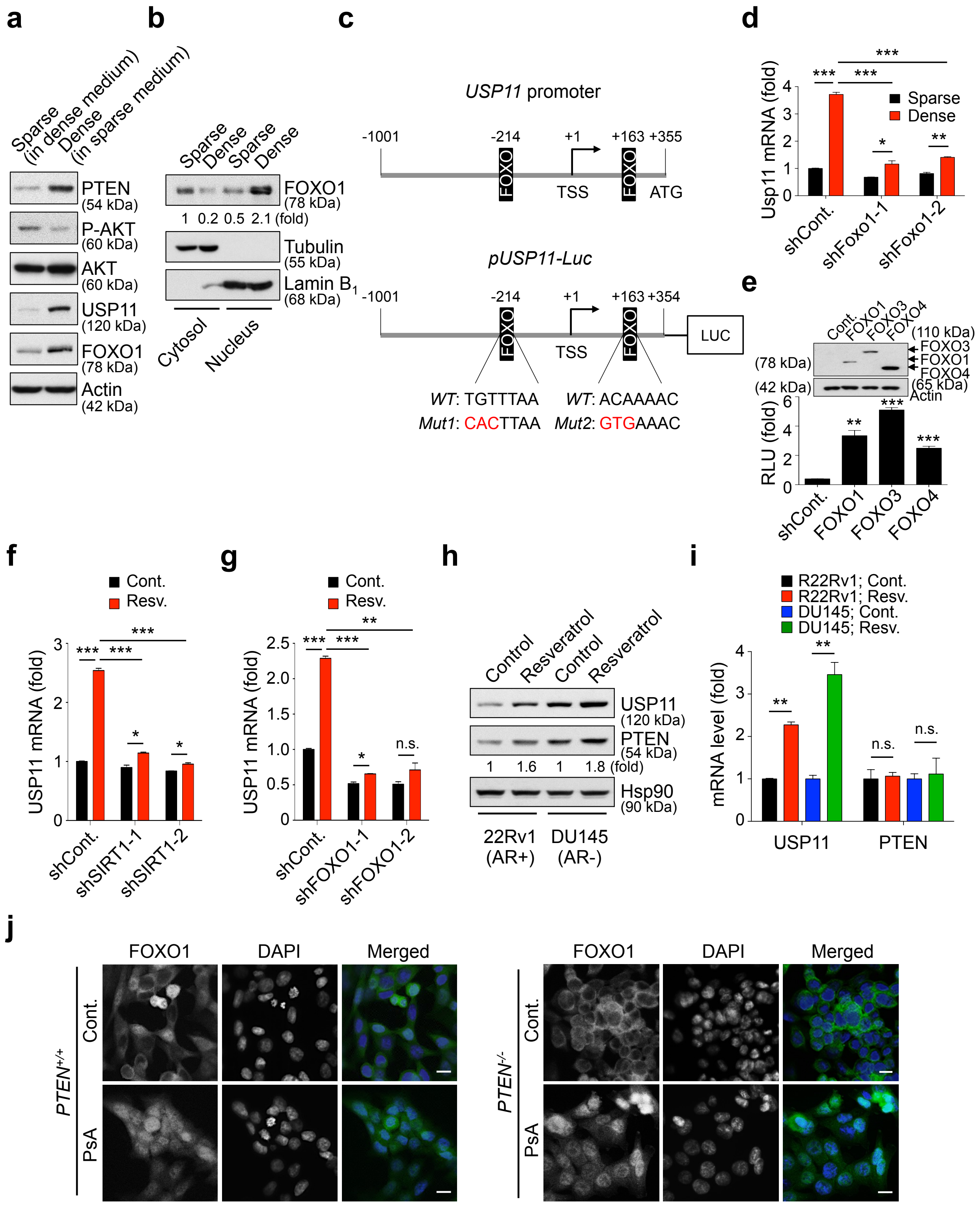
a
b
c
d
Sparse (in dense medium)
Dense (in sparse medium)
PTEN (54 kDa)
P-AKT (60 kDa)
AKT (60 kDa)
USP11 (120 kDa)
FOXO1 (78 kDa)
Actin (42 kDa)
Sparse Dense Sparse Dense
FOXO1 (78 kDa)
1 0.2 0.5 2.1 (fold)
Tubulin (55 kDa)
Lamin B1 (68 kDa)
Cytosol
Nucleus
USP11 promoter
-1001 -214 +1 +163 +355
FOXO
TSS
ATG
pUSP11-Luc
-1001 -214 +1 +163 +354
LUC
WT: TGTTTAA
Mut1: CACTTAA
WT: ACAAAAC
Mut2: GTGAAAC
Usp11 mRNA (fold)
Sparse
Dense
shCont.
shFoxo1-1
shFoxo1-2
e
Cont. FOXO1 FOXO3 FOXO4
(110 kDa) FOXO3
(78 kDa) FOXO1
FOXO4
(42 kDa) (65 kDa)
Actin
RLU (fold)
shCont.
FOXO1
FOXO3
FOXO4
f
Cont.
Resv.
USP11 mRNA (fold)
shCont.
shSIRT1-1
shSIRT1-2
g
Cont.
Resv.
USP11 mRNA (fold)
shCont.
shFOXO1-1
shFOXO1-2
n.s.
h
Control Resveratrol Control Resveratrol
USP11 (120 kDa)
PTEN (54 kDa)
1 1.6 1 1.8 (fold)
Hsp90 (90 kDa)
22Rv1 (AR+)
DU145 (AR-)
i
R22Rv1; Cont.
R22Rv1; Resv.
DU145; Cont.
DU145; Resv.
mRNA level (fold)
USP11
PTEN
n.s.
j
FOXO1 DAPI Merged
PTEN+/+
PTEN-/-
Cont.
PsA

Supplementary Figure 8. FOXO upregulates *USP11*

(**a**) Lysates from sparse- and dense–confluent MEFs cultured in dense- and sparse–conditioned media, respectively, were subjected to immunoblotting (IB).

(**b**) Cytosolic and nuclear fractionation of FOXO1 protein in sparse- and dense–confluent MEFs. Hsp90 and Lamin B_1 serve as controls.

(**c**) Schematic diagrams representing the 5'-regulatory region of human *USP11* gene and the luciferase (LUC) reporter of the *USP11* promoter (*pUSP11-Luc*) harboring wild-type or mutated sequences of putative FOXO-binding sites.

(**d**) Total RNAs from sparse- and dense–confluent MEFs expressing two independent Foxo1 shRNAs were subjected to RT-qPCR.

(**e**) Luciferase reporter analysis of the *USP11* promoter in NIH-3T3 cells overexpressing FOXO1, FOXO3 or FOXO4.

(**f, g**) Total RNAs from DU145 cells expressing two independent SIRT1 (**f**) or FOXO1 (**g**) shRNAs treated with 25 μM resveratrol for 16 h were subjected to RT-qPCR. n = 3.

(**h, i**) Lysates and total RNAs from androgen receptor (AR)-positive 22Rv1 and AR-negative DU145 human prostate cancer cells treated with 25 μM resveratrol for 16 h were subjected to immunoblotting (**a**) and RT-qPCR (**b**). n = 3.

(**j**) Immunofluorescence analysis of FOXO1 in $PTEN^{+/+}$ and $PTEN^{-/-}$ HCT116 cells treated with 5 μM psammaplysene A (PsA) for 24 h. Scale bars, 10 μm.

Error bars represent +/- SEM. *p* value was determined by Student's *t* test (n.s., non-significant; *$p<0.05$; **$p<0.01$; ***$p<0.001$).

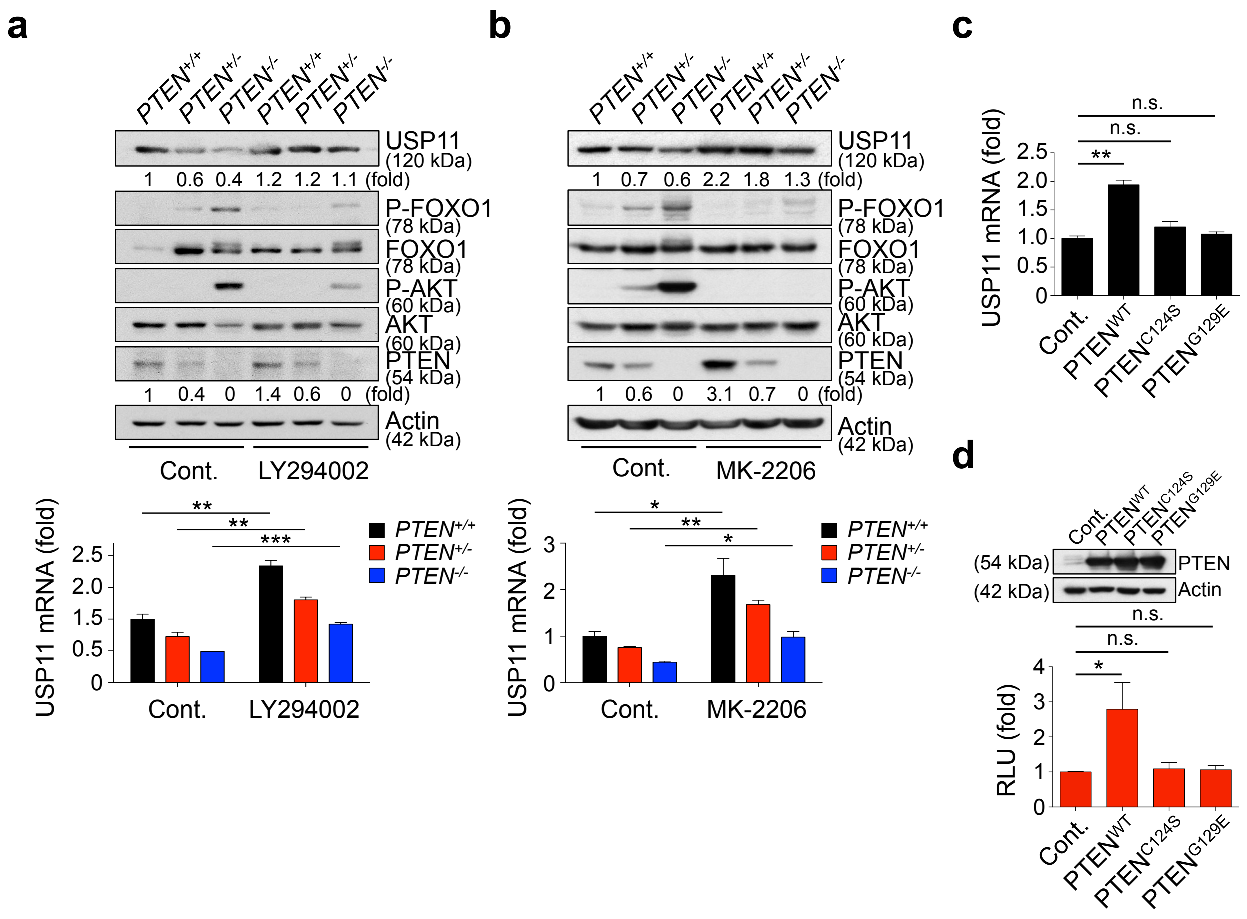

Supplementary Figure 9. The PI3K/AKT pathway regulates the USP11-PTEN axis

(**a**) Lysates and total RNAs from *PTEN*$^{+/+}$, *PTEN*$^{+/-}$ and *PTEN*$^{-/-}$ HCT116 cells treated with 10 μM LY294002 for 8 h were subjected to IB (top) and RT-qPCR (bottom). n = 3.

(**b**) Lysates and total RNAs from *PTEN*$^{+/+}$, *PTEN*$^{+/-}$ and *PTEN*$^{-/-}$ HCT116 cells treated with 1 μM MK-2206 for 24 h were subjected to IB (top) and RT-qPCR (bottom). n = 3.

(**c**) Total RNAs from *PTEN*$^{-/-}$ HCT116 cells transfected with wild-type (WT) or phosphatase-inactive PTEN^{C124S} and PTEN^{G129E} to RT-qPCR. n = 3.

(**d**) Luciferase reporter analysis of the *USP11* promoter in *PTEN*$^{-/-}$ HCT116 cells transfected with PTEN^{WT}, PTEN^{C124S} or PTEN^{G129E}. n = 3.

Error bars represent +/- SEM. *p* value was determined by Student's *t* test (*$p<0.05$; **$p<0.01$; ***$p<0.001$).

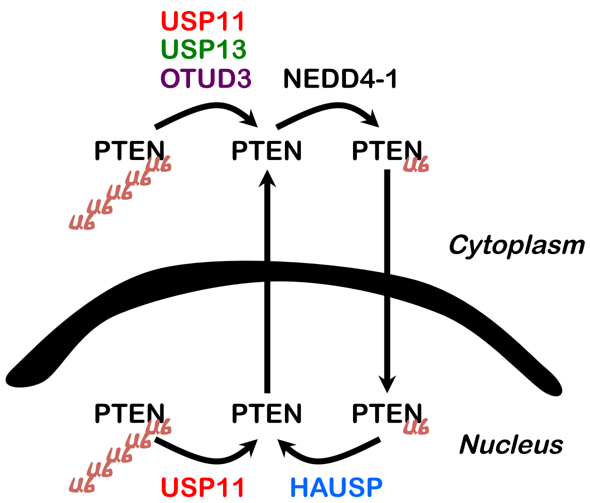

Supplementary Figure 10. A proposed model for PTEN deubiquitination by USP11, HAUSP, USP13 and OTUD3.

USP11 stabilizes nuclear PTEN by de-polyubiquitination, whereas HAUSP induces de-monoubiquitination and nuclear export of PTEN. In cytoplasm, USP11, USP13 and OTUD3 maintain PTEN protein stability by promoting its de-polyubiquitination.

Supplementary Figure 11. Full images of immunoblots

Full unprocessed images and signals detected by immunoblotting, with the regions used in the corresponding main display items indicated by red frames.

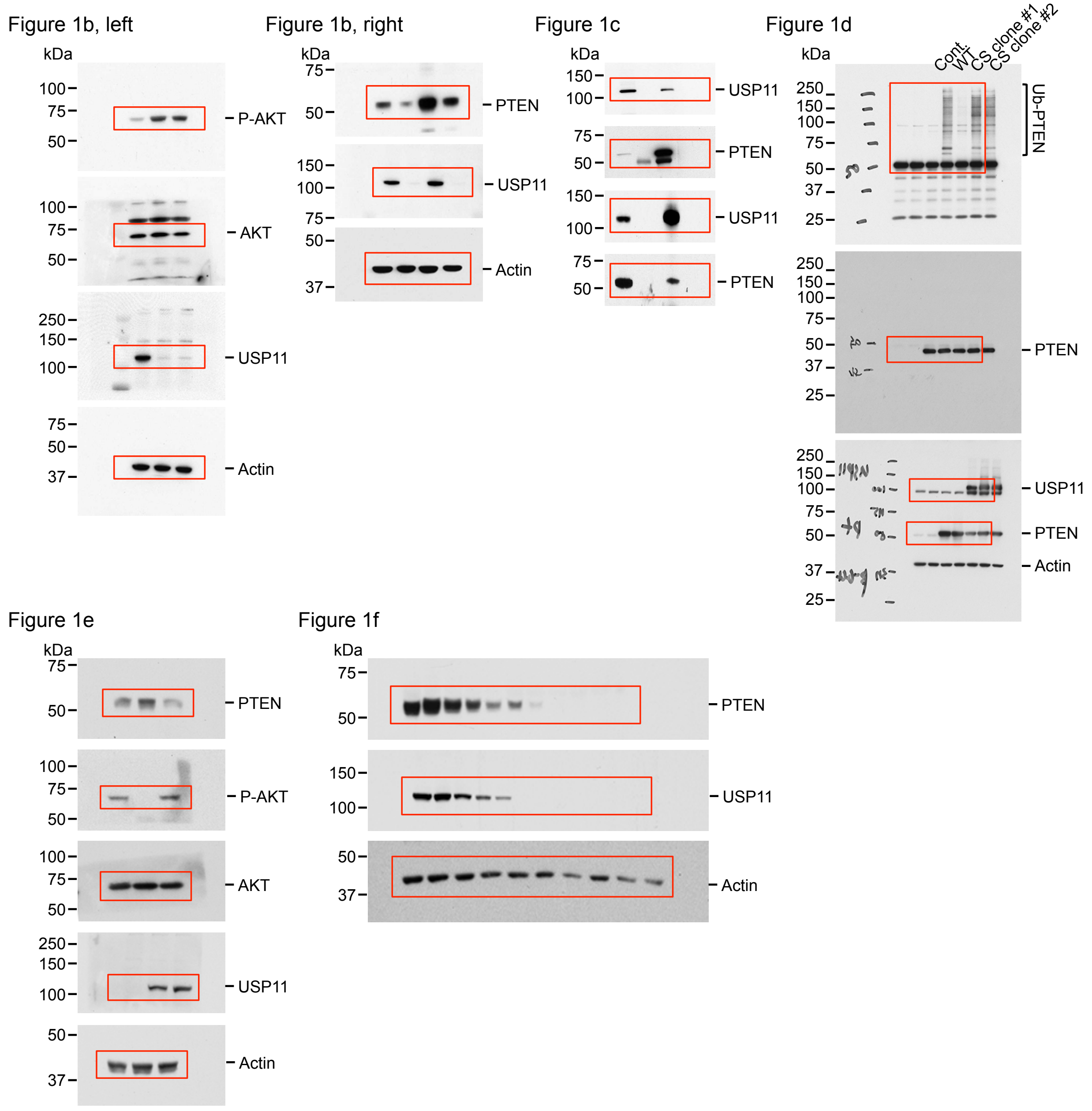

Figure 1h

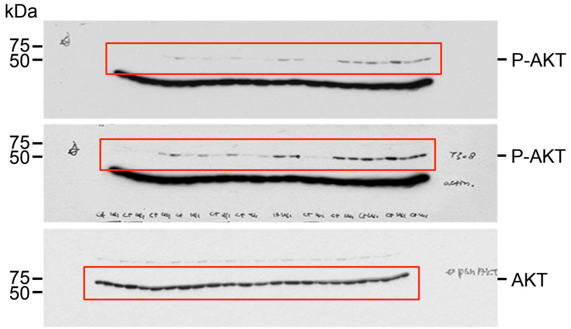

Figure 1h – cont'd

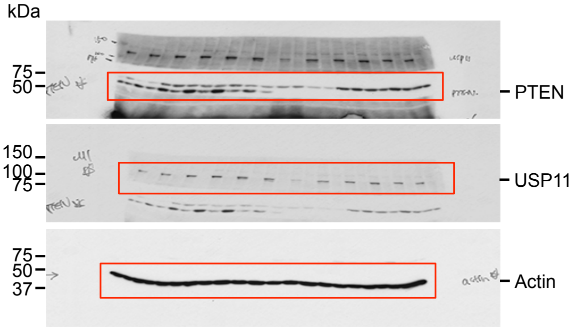

Figure 2d

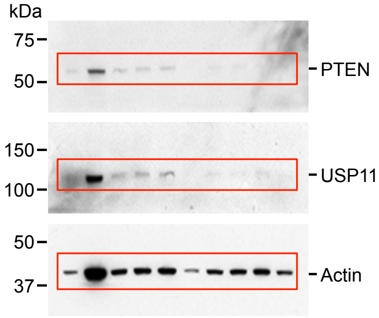

Figure 2i

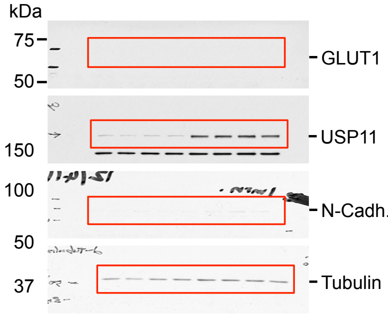

Figure 2i – cont'd

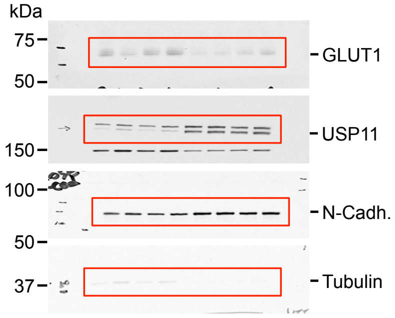

Figure 3a

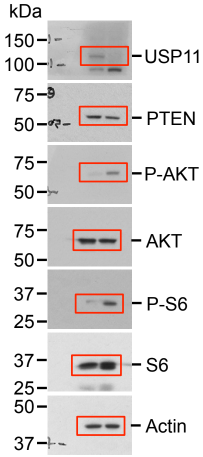

Figure 4a

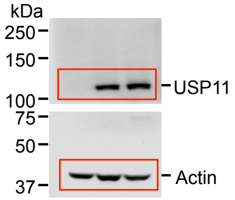

Figure 4d

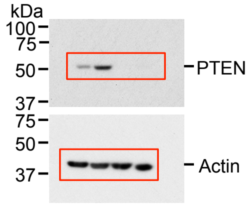

Figure 4g

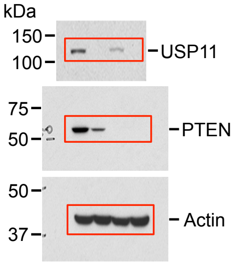

Figure 6a, left

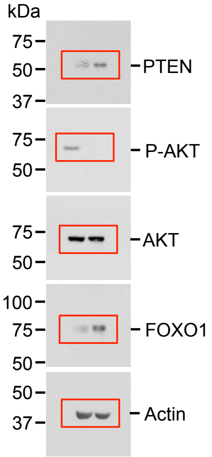

Figure 6a, right & 6e

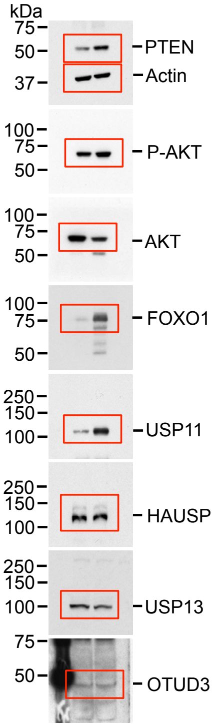

Figure 6c

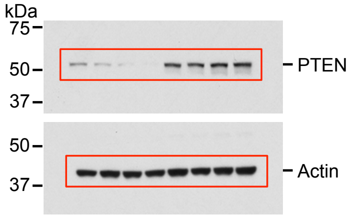

Figure 6d

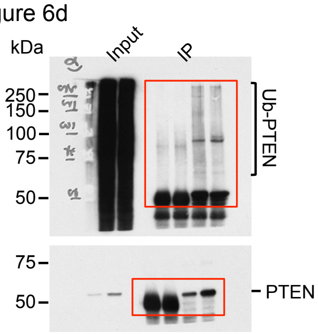

Figure 6g

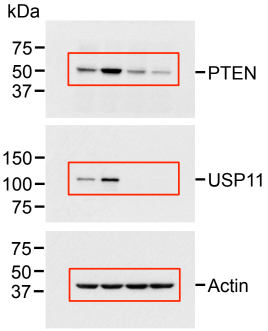

Figure 6h

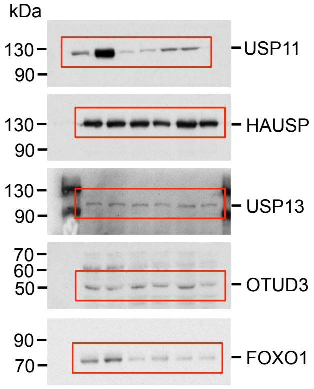

Figure 6i

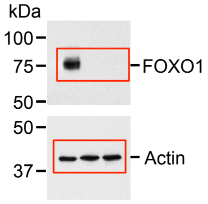

Figure 6j

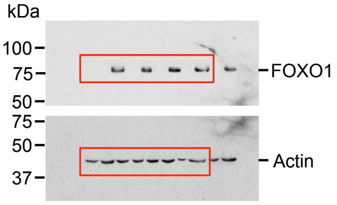

Figure 6l

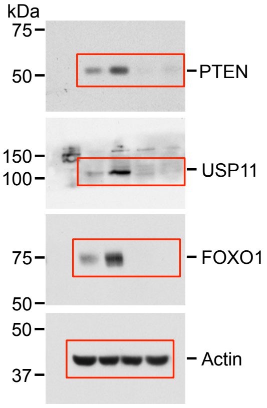

Figure 7a

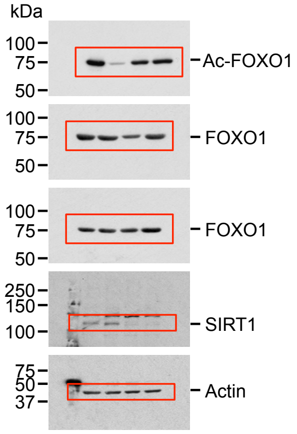

Figure 7b

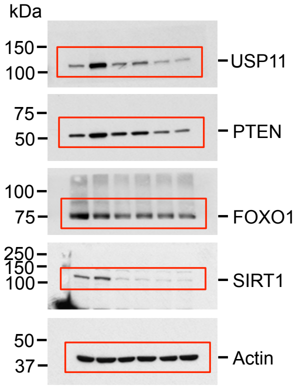

Figure 7c

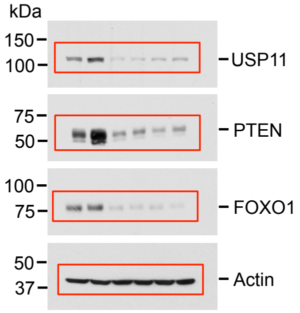

Figure 7d

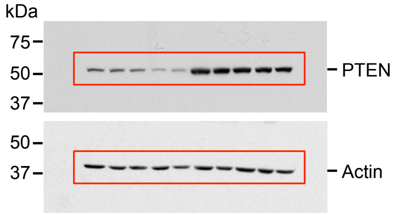

Figure 7f

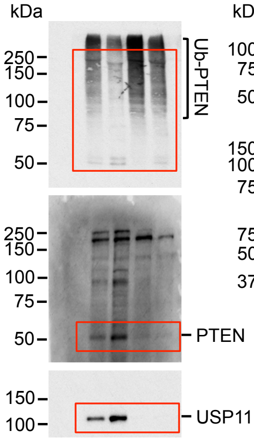

Figure 7g

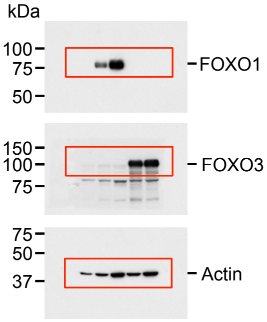

Figure 7i

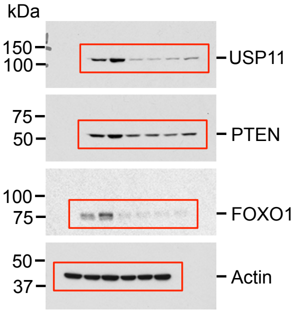

Figure 8a

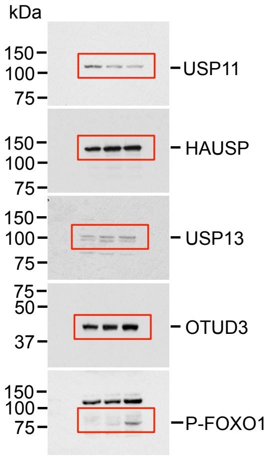

Figure 8a – cont'd

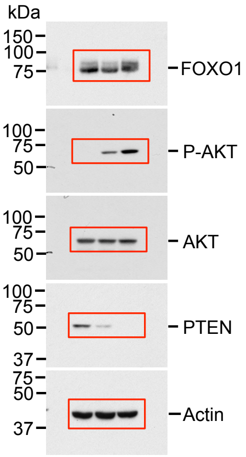

Figure 8e

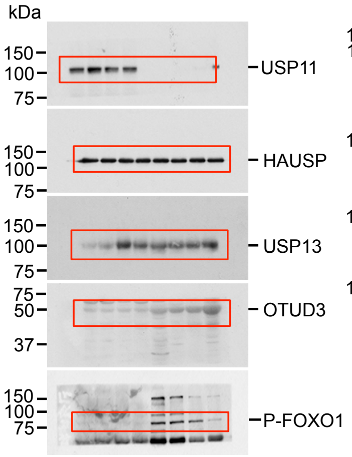

Figure 8e – cont'd

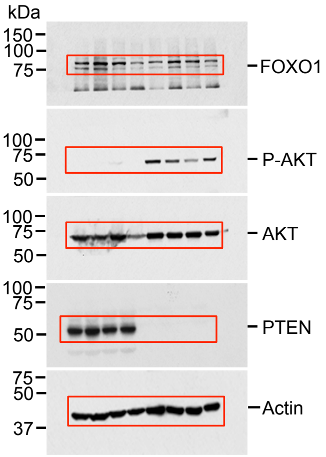

Figure 8f

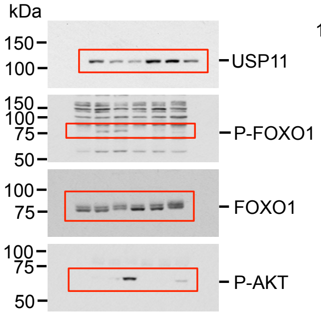

Figure 8f – cont'd

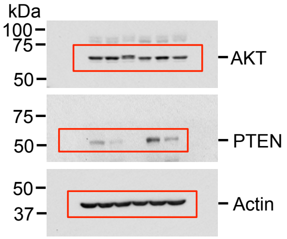

Figure 8g

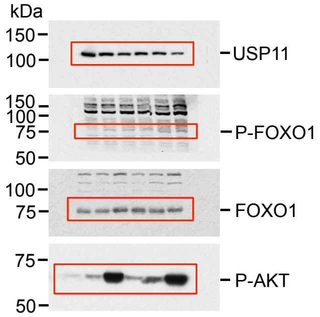

Figure 8g – cont'd

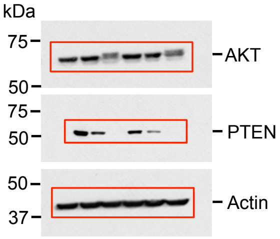

Figure 8h

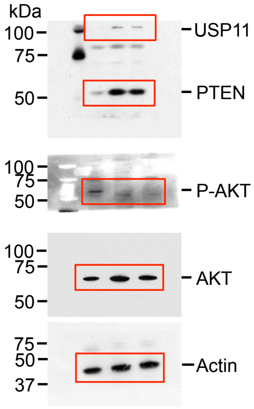

Figure 8i

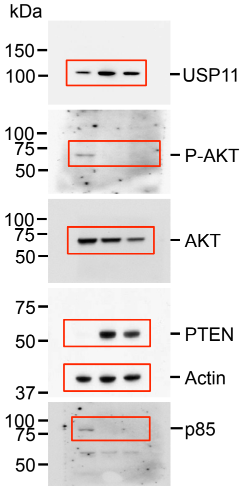

Figure 8j

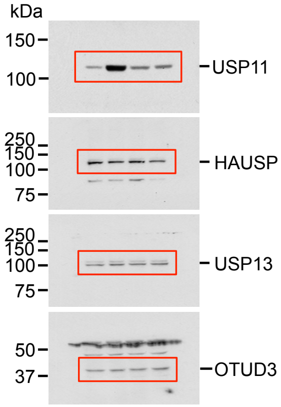

Figure 8j – cont'd

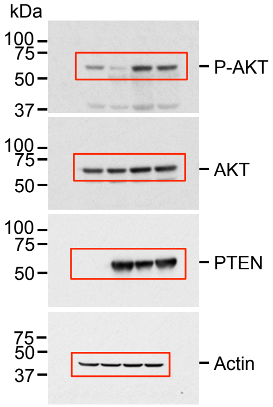

Suppl Fig 1c

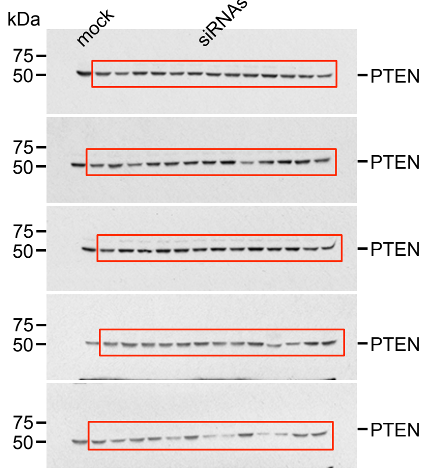

Suppl Fig 1c – cont'd

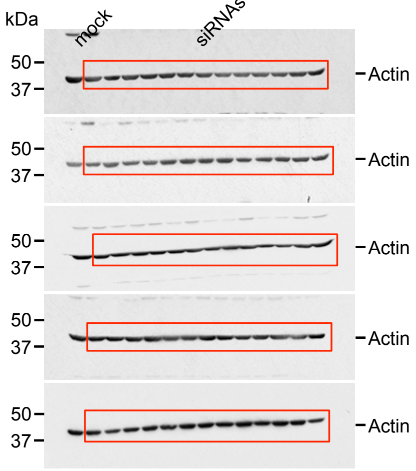

Suppl Fig 2b

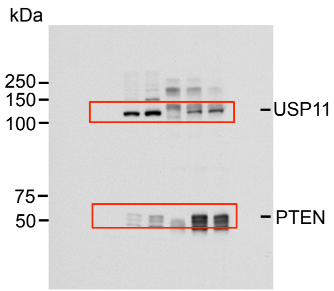

Suppl Fig 2c

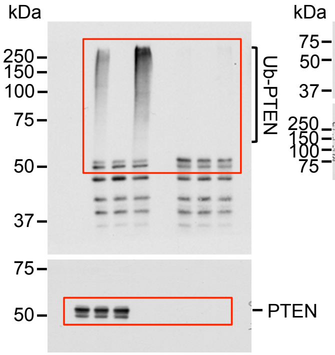

Suppl Fig 2c – cont'd

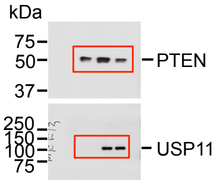

Suppl Fig 2d

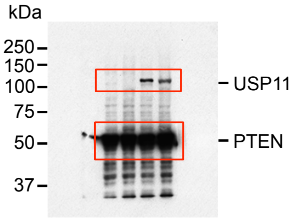

Suppl Fig 2e

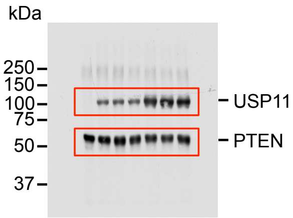

Supple Fig 3a

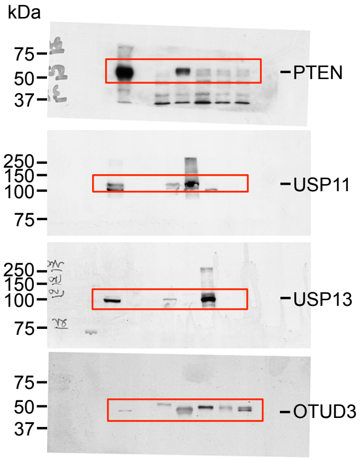

Suppl Fig 3b

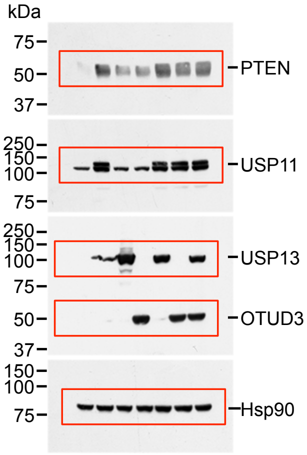

Suppl Fig 3c

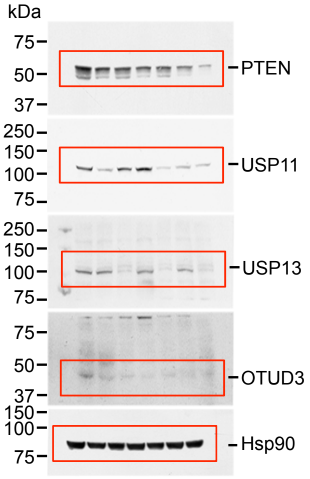

Suppl Fig 3d

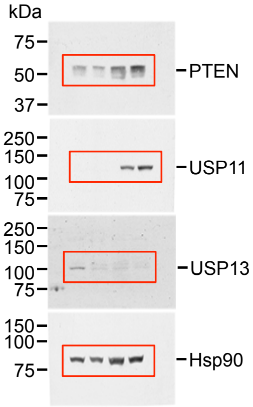

Suppl Fig 3e

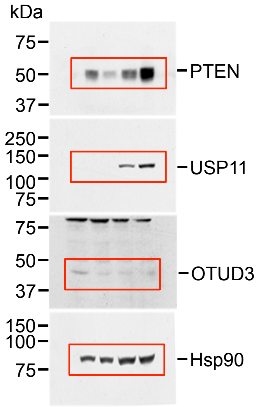

Suppl Fig 3f

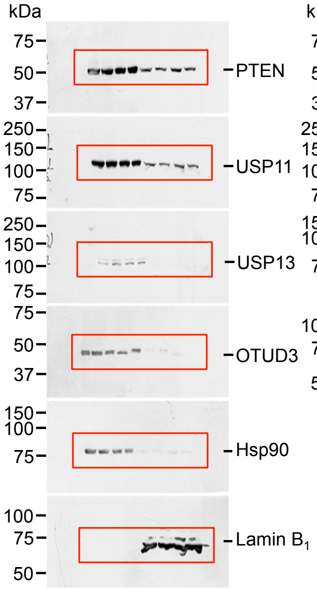

Suppl Fig 3g

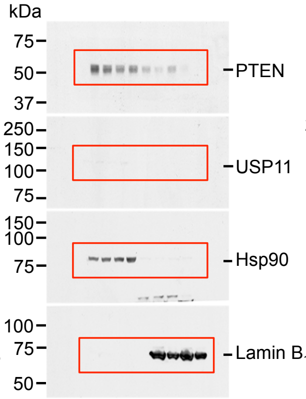

Suppl Fig 3h

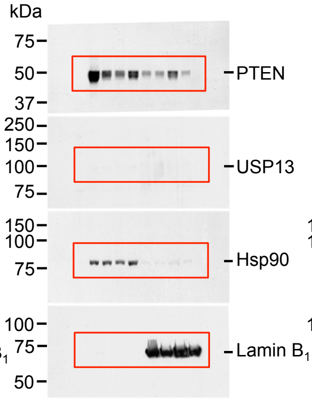

Suppl Fig 3i

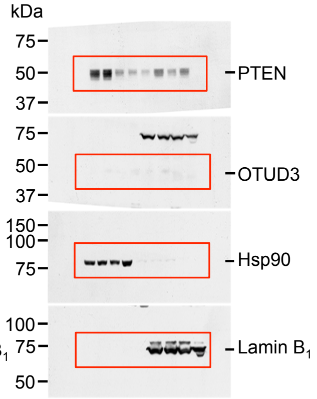

Supple Fig 5b

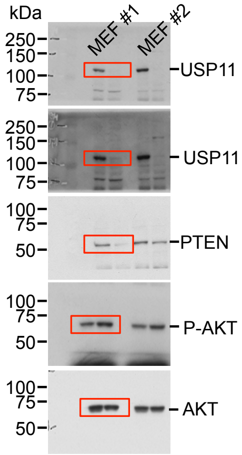

Supple Fig 5b – cont'd

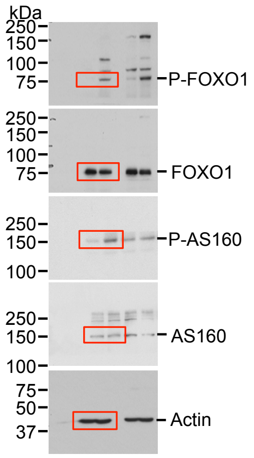

Suppl Fig 3j

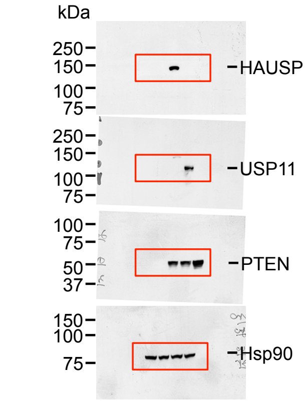

Suppl Fig 3j – cont'd

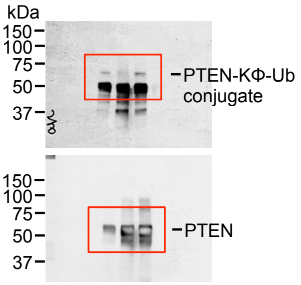

Suppl Fig 5d

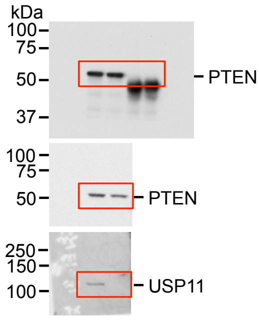

Suppl Fig 5f

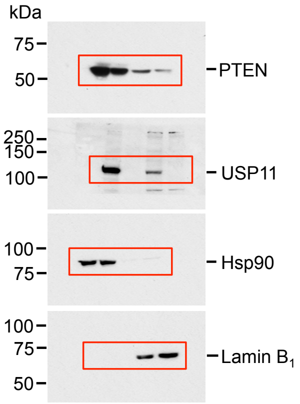

Suppl Fig 7a

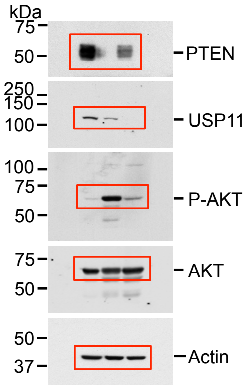

Suppl Fig 7d

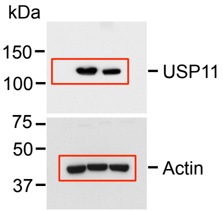

Suppl Fig 8a

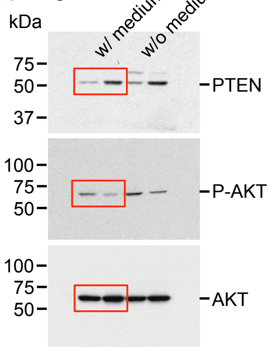

Suppl Fig 8a – cont'd

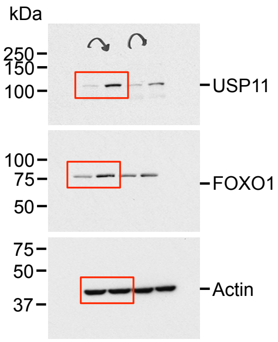

Suppl Fig 8b

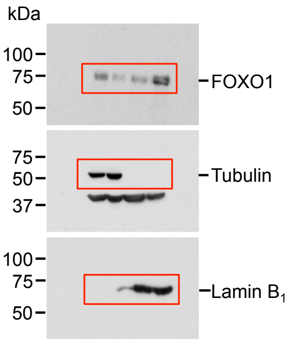

Suppl Fig 8e

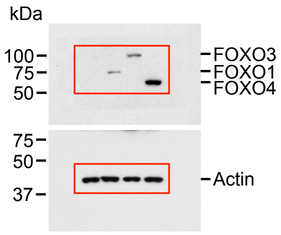

Suppl Fig 8h

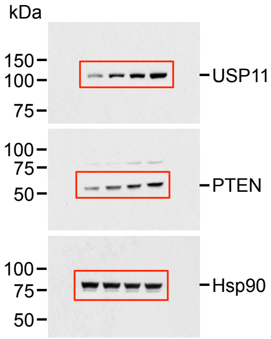

Suppl Fig 9a

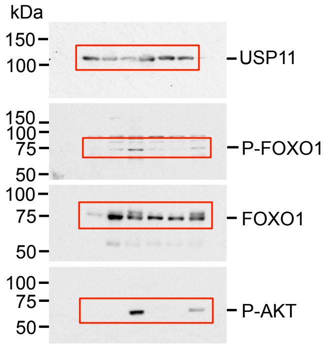

Suppl Fig 9a – cont'd

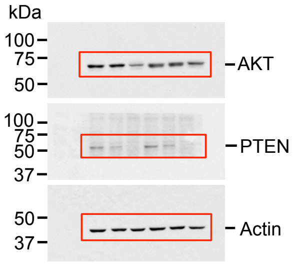

Suppl Fig 9b

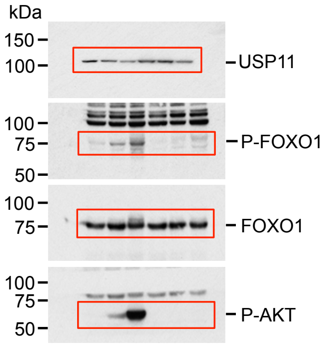

Suppl Fig 9b – cont'd

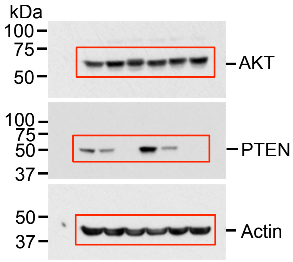

Suppl Fig 9d

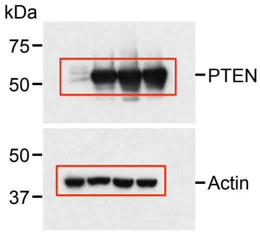

Supplementary Table 1. List of siRNAs and shRNAs

siRNA and shRNA sequences

**Target Gene**	**Sequence**
siRNA	
Control siRNA pool	(1) UGGUUUACAUGUCGACUAA<br>(2) UGGUUUACAUGUUGUGUGA<br>(3) UGGUUUACAUGUUUUCUGA<br>(4) UGGUUUACAUGUUUUCCUA
Human USP11 siRNA pool	(1) GCGCACAGCUGCAUGUCAU<br>(2) GAGAAGCACUGGUAUAAGC<br>(3) GGACCGUGAUGAUAUCUUC<br>(4) GAAGAAGCGUUACUAUGAC
shRNA	
Control shRNA	CCGGGCGCGATAGCGCTAATAATTTCTCGAGAAATTATTAGCGCTATCGCGCTTTTT
Human USP11 shRNA_1	CCGGCGGCACAATGATTTGGGCAAACTCGAGTTTGCCCAAATCATTGTGCCGTTTTT
Human USP11 siRNA_2	CCGGCCGATTCTATTGGCCTAGTATCTCGAGATACTAGGCCAATAGAATCGGTTTTT
Mouse Usp11 shRNA_1	CCGGGCAGCCTATGTCTTGTTCTATCTCGAGATAGAACAAGACATAGGCTGCTTTTTG
Mouse Usp11 shRNA_2	CCGGCCTACTATGGTCTGATACTTTCTCGAGAAAGTATCAGACCATAGTAGGTTTTTG
Human FOXO1 shRNA_1	CCGGATCTACGAGTGGATGGTCAACTCGAGTTGACCATCCACTCGTAGATCTTTTTG
Human FOXO1 shRNA_2	CCGGGCCGGAGTTTAGCCAGTCCAACTCGAGTTGGACTGGCTAAACTCCGGCTTTTTG
Mouse FoxO1 shRNA_1	CCGGCCGCCAAACACCAGTCTAAATCTCGAGATTTAGACTGGTGTTTGGCGGTTTTTG
Mouse FoxO1 shRNA_2	CCGGCCCGTGAAGACACCTTTACAACTCGAGTTGTAAAGGTGTCTTCACGGGTTTTTG
Human SIRT1 shRNA_1	CCGGCAGGTCAAGGGATGGTATTTACTCGAGTAAATACCATCCCTTGACCTGTTTTTG
Human SIRT1 shRNA_2	CCGGCATGAAGTGCCTCAGATATTACTCGAGTAATATCTGAGGCACTTCATGTTTTTTG
Mouse Sirt1 shRNA_1	CCGGAGTGAGACCAGTAGCACTAATCTCGAGATTAGTGCTACTGGTCTCACTTTTTTG
Mouse Sirt1 shRNA_2	CCGGGCCATGTTTGATATTGAGTATCTCGAGATACTCAATATCAAACATGGCTTTTTG

Supplementary Table 2. List of primers used for real-time qPCR

Real-time qPCR primer sequences

Gene symbol	*Accession no.	Forward primer	Reverse primer	Amplicon size (bp)
Primers for human				
USP11	NM_004651	GGACATCGTGGTTCCTGTCT	GACGATGGGGATGAGAAAGA	216
PTEN	NM_000314	GTTTACCGGCAGCATCAAAT	ACTGTGCACTAAAGTGGGGG	197
β-Actin	NM_001101	GGACTTCGAGCAAGAGATGG	TGTGTTGGGGTACAGGTCTTTG	229
MMP9	NM_004994	GGGACGCAGACATCGTCATC	TCGTCATCGTCGAAATGGGC	139
MMP10	NM_002425	TCAGTCTCTCTACGGACCTCC	CAGTGGGATCTTCGCCAAAAATA	183
MMP11	NM_005940	GGGTGTACGACGGTGAAAAG	GTGGAAACGCCAGTAGTCCC	149
MMP12	NM_002426	GATCCAAAGGCCGTAATGTTCC	TGAATGCCACGTATGTCATCAG	86
MMP13	NM_002427	CCAGACTTCACGATGGCATTG	GGCATCTCCTCCATAATTTGGC	137
MMP14	NM_004995	CATCTGTGACGGGAACTTTGA	GGCAGTGTTGATGGACGCA	163
MMP16	NM_005941	ATGCAGCAGTTCTATGGCATT	CTGGTCAGGTACACCGCATC	93
Primers for mouse				
Usp11	NM_145628	ATGTGCCACAGCTGACAGAG	CAAGAACAAGGTTGGCCATT	180
Pten	NM_008960	TGGATTCGACTTAGACTTGACCT	CTGAGAGACATTATGACACCGC	180
β-Actin	NM_007393	TGAGAGGGAAATCGTGCGTGAC	CCGCAGACTGTAGCACATAAAA	186

*GenBank is available at the URL: http://www.ncbi.nlm.nih.gov/genbank/

Supplementary Table 3. List of primers used for cloning and mutagenesis

Primer sequences used for cloning and mutagenesis

Target Gene	Forward primer	Reverse primer
Cloning		
pGEX5X-1 USP11	CGGAATTCATGGCA GTAGCCCCGCGA CTG	CCCGCTCGAGTCAATTAACATCCATGAACTCAGA
pMSCV USP11	CCCGCTCGAGGCCACCATGGCA GTAGCCCCGCGAC	CCGTTAACTCAATTAACATCCATGAACTCAGAGCTGG
Site-directed mutagenesis primers		
USP11 ^{C318S}	CAATCTGGGCAACACGAGCTTCATGAACTCGGC	GCCGAGTTCATGAAGCTCGTGTTGCCCAGATTG
USP11 Promotor^{TGT788CAC} (Mut1)	GAGAATGTTTGGCCTGGCACTTAAGGTCACAGCTCTG	CAGAGCTGTGACCTTAAGTGCCAGGCCAAACATTCTC
USP11 Promotor^{ACA1170GTG} (Mut2)	CTGATCAGGCTAACAAAAGTGCTGCTCCCAGGGTAGGAG	CTCCTACCCTGGGAGCAGCACTTTTGTTAGCCTGATCAG

Supplementary Table 4. List of primers used for ChIP qPCR

ChIP qPCR sequences

Primer region	Forward primer	Reverse primer	Amplicon size (bp)
mouse Usp11			
-2kb region	TGGAAAGCAATTGAGGTTGA	TGTGAGGCTACATTGGCTGT	100
promoter region	TCCTGAGCTGATAGCATGTGA	CGGCAGACCTAGTTCCAGAG	79
+2kb region	GGGATCCACAGCTTTTAACAAA	AACCCGGGGAGCAGTAAAT	90
human USP11			
-2kb region	TAAGCAGAAGAGGGGGTGAG	CCCACCACTGGGCTTTAGTA	91
promoter region	GAGAATGTTTGGCCTGGTGT	TGGTGGAGCTGCAGTTTAGA	74
+2kb region	AATGGAAACGTCGGTGAAAT	TCACAGCCACAATGATCACA	77
