# Supplementary material for: PTEN self-regulates through USP11 via the PI3K-FOXO pathway to stabilize tumor suppression
Source: Nat Commun. 2019 Feb 7;10:636. doi: 10.1038/s41467-019-08481-x (PMC6367354; doi:10.1038/s41467-019-08481-x)
Supplement: Supplementary file 3 — Description of Additional Supplementary Information [file 41467_2019_8481_MOESM3_ESM.pdf]

Description of Additional Supplementary Files

File Name: Supplementary Data 1
Description: Information, including gene accession number, targeting region and sequence, for a siRNA or shRNA library targeting members of mouse deubiquitinating enzyme group.
