# Supplementary material for: PTEN self-regulates through USP11 via the PI3K-FOXO pathway to stabilize tumor suppression
Source: Nat Commun. 2019 Feb 7;10:636. doi: 10.1038/s41467-019-08481-x (PMC6367354; doi:10.1038/s41467-019-08481-x)
Supplement: Supplementary file 5 — Reporting Summary [file 41467_2019_8481_MOESM5_ESM.pdf]

nature research

Corresponding author(s): NCOMMS-17-28545D

# Reporting Summary

Nature Research wishes to improve the reproducibility of the work that we publish. This form provides structure for consistency and transparency in reporting. For further information on Nature Research policies, see Authors & Referees and the Editorial Policy Checklist.

## Statistical parameters

When statistical analyses are reported, confirm that the following items are present in the relevant location (e.g. figure legend, table legend, main text, or Methods section).

| n/a | Confirmed | |
|---|---|---|
| ☐ | ☒ | The exact sample size (*n*) for each experimental group/condition, given as a discrete number and unit of measurement |
| ☐ | ☒ | An indication of whether measurements were taken from distinct samples or whether the same sample was measured repeatedly |
| ☐ | ☒ | The statistical test(s) used AND whether they are one- or two-sided<br>*Only common tests should be described solely by name; describe more complex techniques in the Methods section.* |
| ☐ | ☒ | A description of all covariates tested |
| ☐ | ☒ | A description of any assumptions or corrections, such as tests of normality and adjustment for multiple comparisons |
| ☐ | ☒ | A full description of the statistics including central tendency (e.g. means) or other basic estimates (e.g. regression coefficient) AND variation (e.g. standard deviation) or associated estimates of uncertainty (e.g. confidence intervals) |
| ☐ | ☒ | For null hypothesis testing, the test statistic (e.g. *F*, *t*, *r*) with confidence intervals, effect sizes, degrees of freedom and *P* value noted<br>*Give P values as exact values whenever suitable.* |
| ☒ | ☐ | For Bayesian analysis, information on the choice of priors and Markov chain Monte Carlo settings |
| ☒ | ☐ | For hierarchical and complex designs, identification of the appropriate level for tests and full reporting of outcomes |
| ☐ | ☒ | Estimates of effect sizes (e.g. Cohen's *d*, Pearson's *r*), indicating how they were calculated |
| ☐ | ☒ | Clearly defined error bars<br>*State explicitly what error bars represent (e.g. SD, SE, CI)* |

*Our web collection on statistics for biologists may be useful.*

## Software and code

Policy information about availability of computer code

| | |
|---|---|
| Data collection | (1) Gen5 data analysis software; (2) iMark reader onboard software; (3) Leica DMI 3000B fluorescence microscope on board software; (4) ImagePro software; (5) Nikon 80i fluorescence microscope on board software; (6) Oxirix Imaging software |
| Data analysis | (1) SPSS V.20.0; (2) GraphPad Prism 6; (3) Oxirix Imaging software |

For manuscripts utilizing custom algorithms or software that are central to the research but not yet described in published literature, software must be made available to editors/reviewers upon request. We strongly encourage code deposition in a community repository (e.g. GitHub). See the Nature Research guidelines for submitting code & software for further information.

## Data

Policy information about availability of data

All manuscripts must include a data availability statement. This statement should provide the following information, where applicable:

- Accession codes, unique identifiers, or web links for publicly available datasets
- A list of figures that have associated raw data
- A description of any restrictions on data availability

A clear statement included in each Figure Legend. In Fig. 5a, b, Stephenson's CaP (ref. 40, n = 97); Taylor's CaP (GEO: GSE21032, n = 179); Liu's BCa (GEO: GSE22820,

n = 176). In Fig. 5c, relapse-free survival (RFS) (n = 618) and overall survival (OS) (n = 241) in human basal-type breast cancer patients (http://kmplot.com/analysis/) (ref 43). All the data sets are publicly placed.

# Field-specific reporting

Please select the best fit for your research. If you are not sure, read the appropriate sections before making your selection.

☒ Life sciences ☐ Behavioural & social sciences ☐ Ecological, evolutionary & environmental sciences

For a reference copy of the document with all sections, see nature.com/authors/policies/ReportingSummary-flat.pdf

# Life sciences study design

All studies must disclose on these points even when the disclosure is negative.

| | |
|---|---|
| Sample size | Used higher than average sample size according to data published by other research groups. Always prepare duplicate or triplicate. All experiments were repeated at least double or three times. For animal studies, sample size was calculated before experiment in order to achieve desired power above 0.8 and significance level, and obtain valid results. |
| Data exclusions | Criteria for exclusion: samples not treated properly; illness or poor conditions due to reasons other than treatment; not within 95% range of normal distribution. All criteria were pre-established. |
| Replication | Used higher than average sample size according to data published by other research groups. Always prepare duplicate or triplicate. All experiments were repeated at least double or three times. A clear statement included in each Figure Legend. |
| Randomization | Samples were randomly selected so equal distributions were obtained. For animal studies, subjects may be excluded initially based on recorded qualitative observations. Animals were then randomized iteratively so equal distributions were obtained . ANOVA results are displayed to ensure similarity between groups. |
| Blinding | Blind grouping in both cell line and animal studies was done whenever it's necessary. |

# Reporting for specific materials, systems and methods

## Materials & experimental systems

| n/a | Involved in the study | |
|---|---|---|
| ☒ | ☐ | Unique biological materials |
| ☐ | ☒ | Antibodies |
| ☐ | ☒ | Eukaryotic cell lines |
| ☒ | ☐ | Palaeontology |
| ☐ | ☒ | Animals and other organisms |
| ☒ | ☐ | Human research participants |

## Methods

| n/a | Involved in the study | |
|---|---|---|
| ☒ | ☐ | ChIP-seq |
| ☒ | ☐ | Flow cytometry |
| ☒ | ☐ | MRI-based neuroimaging |

## Antibodies

Antibodies used

Catalog numbers are provided for all antibodies.

• PI(3,4,5)P3 antibody (1:200; Z-P345, Echelon Biosciences)
Braccini, L. et al. PI3K-C2γ is a Rab5 effector selectively controlling endosomal Akt2 activation downstream of insulin signalling. Nat. Commun. 2015 Jun 23;6:7400.

• PTEN (1:100 for IF, 1 :200 for IHC; ABM-2052, Cascade)
Song, MS. et al. The deubiquitinylation and localization of PTEN are regulated by a HAUSP-PML network. Nature 2008 455(7214):813-7.

• FOXO1 (1:200 for IF 1: 800 for IHC; ab52857, abcam)
Shao D et al. A functional interaction between Hippo-YAP signalling and FoxO1 mediates the oxidative stress response. Nat Commun 5:3315 (2014).
Arvatz G et al. Post-transcriptional regulation of heparanase gene expression by a 3' AU-rich element. FASEB J 24:4969-76 (2010).

• FOXO1 antibody (1:50 for Chip assay; ab39670, Abcam)
Ouyang W et al. Novel Foxo1-dependent transcriptional programs control T(reg) cell function. Nature 491:554-9 (2012).

- Anti-PTEN (9559), anti-pAKT (pS473; 9271), anti-P-S235/236-S6 (2211), anti-S6 (2217) from Cell Signaling. Anti-Hsp90 (H1775) from sigma, anti-Lamin B1 (ab16048) were from Abcam.
Garcia-Coa et al. Systemic elevation of PTEN induces a tumor suppressive metabolic state. Cell. 2012 Mar 30;149(1):49-62.

- anti-P-AKT (T308, 2965), anti-AKT (pan; 4685), anti-FOXO1 (2880) from Cell Signaling.
Guertin D et al. mTOR Complex 2 Is Required for the Development of Prostate Cancer Induced by Pten Loss in Mice. Cancer Cell. 2009 Feb 3;15(2):148-59.

- anti-P-FOXO1 (pS256; 9461) from Cell Signaling.
Sekine et al. Foxo1 links insulin signaling to C/EBPα and regulates gluconeogenesis during liver development. EMBO J. 2007 Aug 8;26(15):3607-15

- anti-P-GSK3α (9331) from Cell Signaling.
Bailly-Maitre, B et al. Hepatic Bax Inhibitor-1 Inhibits IRE1α and Protects from Obesity-associated Insulin Resistance and Glucose Intolerance. J Biol Chem. 2010 Feb 26;285(9):6198-207.

- Anti-GSK3α/β (368662) was from Calbiochem.
Mussmann, R et al. Inhibition of GSK3 Promotes Replication and Survival of Pancreatic Beta Cells. J Biol Chem. 2007 Apr 20;282(16):12030-7

- anti-Cleaved PARP (9546) , anti-Cleaved Caspase 3(9664) from Cell Signaling.
Premkumar, D et al. Bortezomib-Induced Sensitization of Malignant Human Glioma Cells to Vorinostat-Induced Apoptosis Depends on Reactive Oxygen Species Production, Mitochondrial Dysfunction, Noxa Upregulation, Mcl-1 Cleavage, and DNA Damage. Mol Carcinog. 2013 Feb; 52(2): 118–133.

- Anti-USP11 (ab109232) from Abcam
Orthwein, A et al. A mechanism for the suppression of homologous recombination in G1 cells. Nature 2015 Dec 17;528(7582):422-6

- anti-USP11 (1:100; KG403, Cosmo Bio)
No reference

- Anti-USP13 (A302-762) from Bethyl Laboratories
Zhang, J. et al. Deubiquitylation and stabilization of PTEN by USP13. Nat. Cell Biol. 2013 15:1486-94

- Anti-OTUD3 (ab107646) from Abcam
Yuan, L. et al. Deubiquitylase OTUD3 regulates PTEN stability and suppresses tumorigenesis. Nat. Cell Biol. 2015 17:1169-81

- Anti-HAUSP (A300-033A) was from Bethyl Laboratories.
Gao, Y. et al. Early adipogenesis is regulated through USP7-mediated deubiquitination of the histone acetyltransferase TIP60. Nat. Commun. 4:2656

- anti-Acetylated lysine (9441) from Cell Signaling.
Siudeja K et al. Impaired Coenzyme A metabolism affects histone and tubulin acetylation in Drosophila and human cell models of pantothenate kinase associated neurodegeneration. EMBO Mol Med. 2011 Dec; 3(12): 755–766.

- anti-N-Cadherin (4061) from Cell Signaling.
Chen, L et al. Trim28 Contributes to EMT via Regulation of E-Cadherin and N-Cadherin in Lung Cancer Cell Lines. PLoS One. 2014; 9(7): e101040.

- anti-Myc (2276) from Cell Signaling.
Tong, A et al. ATM and ATR Signaling Regulate the Recruitment of Human Telomerase to Telomeres. Cell Reports. 15 Nov 24;13(8):1633-46

- anti-Actin (A2228), anti-α-Tubulin(T8203) were from Sigma.
Hofling, A et al. Manipulation of pro-inflammatory cytokine production by the bacterial cell-penetrating effector protein YopM is independent of its interaction with host cell kinases RSK1 and PRK2. Virulence. 2014 Oct 1; 5(7): 761–771.

- anti-Flag (F1804) were from Sigma.
Li, S et al.Thioredoxin 2 Is a Novel E2-Interacting Protein That Inhibits the Replication of Classical Swine Fever Virus. J. Virol. August 2015 vol. 89 no. 16 8510-8524

- anti-Glut1 (ab40084) from abcam.
Banerjee, S et al. SGLT1, a Novel Cardiac Glucose Transporter, Mediates Increased Glucose Uptake in PRKAG2 Cardiomyopathy. J Mol Cell Cardiol. 2010 October; 49(4): 683–692.

- anti-SIRT1 (sc-15404) were from Santa Cruz.
Karaca, E et al. Oxygen-dependent acetylation and dimerization of the corepressor CtBP2 in neural stem cells. Experimental Cell Research. 2015 Mar 1;332(1):128-35.

- Anti-Poly-ubiquitylated protein (FK1) was from Biomol .
Huang, L et al. Cutting Edge: Selective Role of Ubiquitin in MHC Class I Antigen Presentation. J Immunol. 2011 Feb 15;186(4):1904-8.

• Anti-HA (MMS-101P) was from Covance.
Tien, CL et al. Snail2/Slug cooperates with Polycomb repressive complex 2 (PRC2) to regulate neural crest development. Development 2015 Feb 15;142(4):722-31.

• anti-FOXO3a (1:800; 12829, Cell Signaling)
Shan Yu et al. Activation of FOXO3a suggests good prognosis of patients with radically resected gastric cancer. Int J Clin Exp Pathol. 2015 Mar 1;8(3):2963-70

• anti-Ki-67 (1:200; RM-9106, Lab Vision)
Rizzi N et al. In vivo imaging of cell proliferation for a dynamic, whole body, analysis of undesired drug effects. Toxicol Sci. 2015 Jun;145(2):296-306

• anti-SV40 T Ag (1:100, 554149, BD Pahrmingen).
Li M et al. Expression of a viral oncoprotein during mammary gland development alters cell fate and function: induction of p53-independent apoptosis is followed by impaired milk protein production in surviving cells. Cell Growth Differ. 1996; 270(1):3-11

Validation

As described above, all the antibodies are selected by publication validation. Otherwise, we have validated their specificity in cell lines expressing shRNAs against the genes or knockout MEFs.

# Eukaryotic cell lines

Policy information about cell lines

Cell line source(s)

293T, DU145, PC3, 22Rv1, NB4, NIH-3T3, U2OS, MCF-10A, MDA-MB-231, MDA-MB-157, HS578T, BT549 and MDA-MB-468 were obtained from ATCC.
HCT116 PTEN+/+, HCT116 PTEN+/-, HCT116 PTEN-/- were purchased from Horizon Discovery.
NB4 cells, a human APL cell line bearing the t(15;17), were a gift of M. Lanotte (Hôpital Saint-Louis, France).

Authentication

Human cell line authentication has been performed at “Characterized Cell Line Facility” at M.D. Anderson Cancer Center - If a human cell line has a known STR profile, the STR profile against a database of known STR profiles is compared; If a human cell line does not have a known STR profile, the core facility compare against all of the samples submitted to the core facility. All unique STR profiles are then added to the database for future M.D. Anderson Cancer Center reference.

Mycoplasma contamination

All cell lines were tested for mycoplasma contamination by both ELISA and PCR genotyping.

Commonly misidentified lines
(See ICLAC register)

None.

# Animals and other organisms

Policy information about studies involving animals; ARRIVE guidelines recommended for reporting animal research

Laboratory animals

Mouse (Mus musculus);
Usp11gt/Y mice, male, 11 weeks; TRAMP and Usp11gt compound mice, male, 20 or 25 weeks;
Swiss Nu/Nu, male, 8-weeks

Wild animals

This study did not involove wild animals.

Field-collected samples

The acquisition, care, housing, use, and disposition of animals in research were in compliance with applicable federal, state, and local, laws and regulations. All animal protocols were approved by UT M.D. Anderson Cancer Center Institutional Animal Care and Use Committee (IACUC).
